# Latent profile analysis of loneliness among elderly people in the community and its relationship with cognitive function

**DOI:** 10.3389/fnagi.2025.1574095

**Published:** 2025-08-07

**Authors:** Lei Yang, Rushi Yang, Tiantian Liu, Jinfeng Wang, Bo Wang, Fengxue Zhao, Yue Zhang, Ping Zhang, Hao Zhang

**Affiliations:** ^1^School of Nursing, Henan Medical University, Xinxiang, Henan, China; ^2^Neurology Intensive Care Unit, East Hospital Affiliated to Tongji University Pudong, Shanghai, China

**Keywords:** older adults, loneliness, community, cognitive function, cognitive impairment, latent profile analysis

## Abstract

**Objectives:**

To explore the latent profiles of loneliness in community-dwelling older adults and to explore the relationship between categories and cognitive functioning to inform targeted interventions.

**Methods:**

A survey was conducted on 658 elderly individuals from 9 communities in Henan Province using the Simplified Loneliness Scale and the Montreal Cognitive Assessment Scale. Mplus8.3 was used for latent profile analysis, and SPSS26.0 software was used to compare the cognitive function differences of elderly people with different types of loneliness.

**Results:**

Prevalence rate of mild cognitive impairment in community-dwelling older adults 31.00% (204/658). The latent profiles of loneliness symptoms in community-dwelling older adults can be categorized into three latent profiles: low loneliness group (54.4%), social loneliness group (24.3%), and emotional loneliness group (21.3%). Community-dwelling older adults in the emotional loneliness group had a higher risk of cognitive impairment compared to the low loneliness group (OR = 1.693, *p* < 0.05).

**Conclusion:**

Three categories of loneliness exist in community-dwelling older adults, with differences in cognitive functioning among community-dwelling older adults with different latent profiles. Community healthcare workers should pay attention to the loneliness of older adults during cognitive function screening, and especially provide psychological counseling to emotionally isolated community-dwelling older adults in order to reduce the risk of cognitive impairment.

## 1 Introduction

In the current era of accelerating population aging, changes in population structure affect various levels of society. The World Health Organization predicts that the global elderly population aged 60 and above will reach 2.1 billion by 2050 (Kidambi and Lee et al., 2020). With the increasing proportion of the elderly population, the mental health issues of the elderly have gradually surfaced, and the mental health issues of the current elderly population are receiving more attention. Loneliness is a subjective psychological state of social and emotional isolation, which is a negative emotional experience (Yu et al., 2023). As age increases, loneliness becomes very common. Surveys in European and American countries have found that the prevalence of loneliness among the elderly ranges from 5 to 43% (Van As et al., 2022). A meta-analysis found that elderly people in Central and Eastern Europe have a higher prevalence of loneliness (Surkalim et al., 2022). However, loneliness not only affects the quality of life of individuals in the elderly population, but numerous studies have found that loneliness often serves as an intermediary for various negative emotions, indirectly affecting the physical and mental health of the body (Lutzman et al., 2021; Zafar et al., 2021). Especially with the increasing number of empty nesters and elderly people living alone, the problem of loneliness among the elderly has become more severe, and their quality of life and subjective well-being have declined (Chokkanathan, 2024). Community healthcare workers should pay more attention to this issue and make corresponding mental health education.

Research has found that loneliness may be associated with the occurrence of mild cognitive impairment, which can directly or indirectly affect the executive and cognitive functions of elderly people through multiple pathways (Lara et al., 2019; Wilson et al., 2021). A cross-sectional study shows that the prevalence of mild cognitive impairment among people over 60 years old in China is 15.5%, and the incidence rate is rising (Jia et al., 2020), however, there are still issues such as low awareness of MCI and immature intervention systems (Pérez et al., 2022). Mild cognitive impairment, as a prodromal stage of Alzheimer’s disease, early prevention can delay the progression of MCI to AD (Cong et al., 2023). In community research, it has been observed that the proportion of individuals developing from MCI to AD is much lower than in clinical settings (Sha et al., 2022). After implementing cognitive management measures for elderly individuals with MCI, some of them were able to restore their cognitive levels to normal (Iraniparast et al., 2022). Therefore, identifying controllable factors that affect cognitive function in elderly individuals with MCI in the community can effectively delay the progression of cognitive impairment to dementia.

Previous studies have mostly adopted a variable centered approach, treating them as homogeneous individuals and paying less attention to whether there is heterogeneity in loneliness levels among elderly people in the community. Although there are various methods to improve loneliness among the elderly, most of them adopt consistent intervention measures to manage the elderly population, without paying attention to the different characteristics among the elderly groups. However, the loneliness emotions of the elderly are complex and exhibit significant group heterogeneity. Therefore, this study adopts the Latent Profile Analysis method to explore the potential subtypes of loneliness among elderly people in the community at the individual center, capturing group heterogeneity. It determines the subgroups to which individuals belong based on the response patterns of heterogeneous groups on external variables, and the objectivity of the model fitting indicators also avoids high heterogeneity within the group caused by subjective classification standards as much as possible, thus capturing the group inequality that cannot be observed in variable centered research (Zheng and Zhang, 2024). Compared with traditional clustering analysis, LPA has stricter criteria for retaining the number of categories and more accurate classification results (Hou and Zhang, 2023). This study aims to explore the potential characteristics of elderly people in communities with different types of loneliness, and analyze the risk relationship between different subgroups of loneliness and cognitive function, providing reference for the development of intervention measures to improve cognitive function in elderly people.

## 2 Materials and methods

### 2.1 Study design and population

This study is a cross-sectional study that used random sampling to study elderly people from 9 communities in a city in Henan Province from July 2021 to October 2022. Inclusion criteria: (1) Age ≥ 60 years old; (2) Residing in the community for at least 12 months; (3) Voluntarily participating in this study; (4) Normal expression and comprehension ability, able to independently complete questionnaire filling or complete the survey through the investigator. Exclusion criteria: (1) Individuals with visual or hearing impairments or accompanied by serious illnesses (such as traumatic brain injury, mental illness, etc.) that affect cognitive testing; (2) Those who have recently suffered from life setbacks. This study distributed 692 survey questionnaires to elderly people in the community, and after excluding 27 invalid questionnaires such as incomplete filling and regular answering, 658 valid questionnaires were finally collected, with an effective rate of 95.08%. This study has been approved by the Ethics Committee of Xinxiang Medical University (XYLL-20220001).

### 2.2 Research instrument

#### 2.2.1 General information questionnaire

The research team independently designed a demographic general information questionnaire, which includes relevant information such as age, gender, education level, marital status, average monthly household income, chronic disease status, and exercise frequency.

#### 2.2.2 Montreal Cognitive Assessment Scale (MoCA)

The MoCA scale is a measurement tool developed by Nasreddine et al. (2005). Based on the MMSE scale, and is used for rapid screening of MCI. The scale includes seven cognitive domains: visual spatial and executive ability, naming ability, attention, language, abstraction ability, delayed recall, and orientation ability. The total score of the scale is 30 points, with ≥ 26 points indicating normal cognition. If the education years are ≤12 years, the total score is increased by 1 point. In this study, the Cronbach’s alpha coefficient of the scale was 0.821.

#### 2.2.3 University of California, Los Angeles Loneliness Scale-8 (ULS-8)

This scale is a simplified version of Russell’s 20 item loneliness scale developed by Hays and DiMatteo (1987), consisting of 8 items, to assess the level of loneliness in older adults. The simplified version of the Loneliness Scale is single dimensional and uses the Likert 4-point rating system, with scores ranging from “never” to “always.” Items 3 and 6 are scored in reverse. The total score is the sum of the scores of 8 items, and the higher the score, the more severe the level of loneliness. The Cronbach’s alpha coefficient of this scale in this study was 0.730.

#### 2.2.4 Geriatric Depression Scale-15 (GDS-15)

This scale is based on the Geriatric Depression Scale and is answered with a “yes” or “no” answer. In Zhang’s research results, it was found that GDS-15 exhibited high internal consistency in all samples (Zhang et al., 2020). This scale contains 15 questions, and the higher the score, the more severe the depressive symptoms. 0–4 points indicate no depressive symptoms, 5–8 points indicate mild depression, 9–11 points indicate moderate depression, and 12–15 points indicate severe depression. The Cronbach’s alpha coefficient of this scale in this study was 0.758.

### 2.3 Data collection

This study used face-to-face questionnaire surveys to collect data, and the results of cognitive impairment were diagnosed by community doctors. During data collection, the researchers followed a uniform protocol, providing consistent verbal instructions to each participant while obtaining informed consent and distributing the survey materials. They systematically detailed the study’s purpose, questionnaire components, and relevant precautions to ensure participant understanding. After the questionnaire was distributed, the research subjects filled it out and collected it on the spot for verification. This study distributed 692 questionnaires and received 658 valid responses, with an effective response rate of 95.08%.

### 2.4 Statistical analysis

The research data was analyzed for potential profiles using Mplus8.3. Starting from the initial model of a single category, gradually increasing the number of potential profiles, testing the adaptability of each model, and ultimately selecting the best potential profile model. Akaike information criteria (AIC), Bayesian information criteria (BIC), and adjusted BIC (aBIC), the smaller the statistical value, the better the fitting effect (Băjenaru et al., 2022). Entropy is used to evaluate the accuracy of classification, when Entropy ≥ 0.8, it indicates that the current classification accuracy is greater than 90%, and the closer the entropy value is to 1, the more accurate the model classification is (Kong and Zhang, 2023). Lo Mendel Rubin (LMR) and Bootstrap based likelihood ratio test (BLRT) are used to compare the fitting differences between different category models. If the BLRT and LMR are *p* < 0.05, it indicates that k category models are better than *k*-1 category models (Zhang et al., 2021). SPSS 26.0 software was used for data analysis. Count data were described using frequency and percentage, and the *χ*^2^ test or Fisher’s exact probability method was used for comparison between multiple groups. The measurement data conforming to normal distribution were expressed as ( $\bar{x}$ ± s), and one-way ANOVA was used for comparison between multiple groups. Quantitative data that do not follow a normal distribution are described using median (*M*) and quartiles (*P*_25_, *P*_75_). The variable assignments are as follows: Gender, male = 1, female = 2; Marital status, widowed = 0, married = 1; Monthly household income, below 2000 RMB = 1, 2000 ~ 4,000 RMB = 2, 4,000 RMB and above = 3; Residential mode, non-living alone = 1, living alone = 2; Chronic disease status, none = 0, one = 1, two or more = 2; Drinking history, no = 0, yes = 1; Interests and hobbies, none = 0, yes = 1; Exercise frequency, almost never = 1, occasionally = 2, often = 3; Cognitive impairment, none = 0, yes = 1. Multivariate analysis was performed by Logistic regression, and the test level *p* < 0.05.

## 3 Results

### 3.1 Common method bias testing

Common Method Bias Testing was applied to all items of the scale. The analysis results showed that the eigenvalues of 9 factors were >1, and the variance explanation rate of the first factor was 14.05%, lower than the critical standard of 40%, indicating that there was no serious common method bias in this study.

### 3.2 General information for the elderly

This study included 658 elderly residents in the community, including 247 males and 411 females; Age range 65–90 (71.76 ± 4.995). Marital status: 534 with spouse and 124 widowed; Family monthly income: 58 cases with income less than 2000 RMB, 369 cases with income between 2000 and 4,000 RMB, and 231 cases with income greater than 4,000 RMB; Residential mode: 57 cases living alone, 601 cases not living alone; Educational level: 84 cases of primary school or below, 208 cases of Middle school, 161 cases of high school, and 205 cases of college and above; Exercise frequency: Almost never 118 cases, occasionally 459 cases, frequently 81 cases. The score of the simplified version of the ULS-8 scale for elderly people in the community is 13.00 (11.00, 16.00) points, the cognitive function score for elderly people in the community is (24.98 ± 2.529) points, and the detection rate of cognitive impairment among elderly people in the community is 31.00% (204/658).

### 3.3 Latent profile analysis of loneliness among elderly people in the community

The study used the scores of 8 items in the simplified version of the ULS-8 scale as external variables to fit the potential profile of loneliness levels among elderly people in the community. This study explored 1–5 potential profile models, and the indicators of the fitted models are shown in Table 1. As the number of profiles increases, the values of AIC, BIC, and aBIC gradually decrease, and the *p* < 0.05 values of LMR and BLRT for each category model are statistically significant. Taking into account various indicators and the practical significance and interpretability of the fitting model, this study believes that the three category model are the optimal model for fitting. The attribution probabilities of the three category models in each category were 94.9, 87.0, and 97.4%, all of which were >85%, indicating the reliability of the potential profile analysis results in this study.

**Table 1:** Latent profiles analysis of loneliness among elderly people in the community and fitting indicators of various models.

Category	AIC	BIC	aBIC	Entropy	LMR (*P*)	BLRT (*P*)	Latent profiles proportion (%)
1	13292.838	13364.665	13313.865				
2	12543.389	12655.620	12576.244	0.803	<0.001	<0.001	0.667/0.333
3	12309.266	12461.899	12353.948	0.851	<0.001	<0.001	0.544/0.243/0.213
4	10677.082	10870.118	10733.592	0.931	0.0034	<0.001	0.144/0.251/0.483/0.122
5	10531.692	10765.131	10600.030	0.937	0.0023	<0.001	0.251/0.456/0.112/0.059/0.122

### 3.4 Latent profile characteristics and naming of loneliness among elderly people in the community

**Figure 1:**
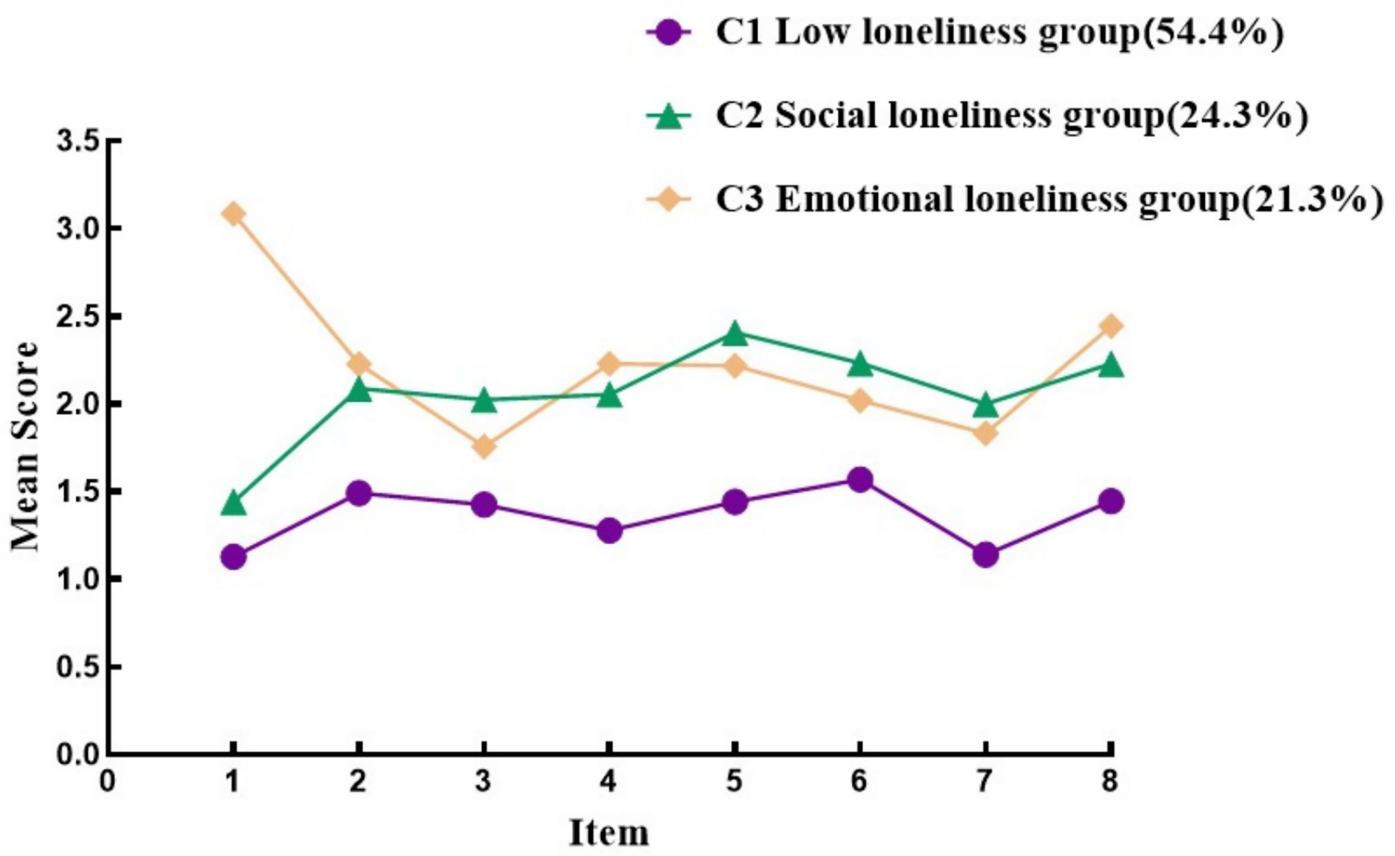
Characteristics distribution of three latent profiles of loneliness among elderly people in the community. Item 1: Lack of companionship. Item 2: Lack of assistance. Item 3: Willing to make friends. Item 4: Feeling neglected. Item 5: Feeling distant from others. Item 6: When there is an emotional need, one can find someone to accompany them. Item 7: Rarely do I feel sad when not communicating with others. Item 8: There are people around, but no one cares.

The scores of each item on the simplified version of the ULS-8 scale for the potential profile of loneliness among elderly people in the community are shown in Figure 1. Among the three categories, 358 elderly people (54.4%) in the first category had low scores in all items, and were named the low loneliness group 11.00 (9.00, 12.00). The second category has a higher average score in items ③, ⑤, ⑥, and ⑦, and the corresponding problem is social activity. Therefore, it is named the social loneliness group 16.00 (15.00, 18.00), with a total of 160 elderly people (24.3%) in this community. The third category has a higher average score in items ①, ②, ④, and ⑧. This question mainly focuses on subjective feelings of loneliness, so it is named the emotional loneliness group 18.00 (15.00, 20.00). There is a total of 140 elderly people (21.3%) in this community.

### 3.5 A univariate analysis of latent profiles on loneliness among elderly people in the community

There were significant differences in gender, marital status, family per capita monthly income, living style, chronic disease status, drinking history, interests and exercise frequency among different potential categories of elderly people in the community (*p* < 0.05) (see Table 2 for details).

**Table 2:** A univariate analysis of latent profiles on loneliness among elderly people in the community [*N* = 658, case (percent, %)].

Variable	Group	Class 1 (*n* = 358)	Class 2 (*n* = 160)	Class 3 (*n* = 140)	*χ*^2^	*P*
Gender	Man	157(43.9)	48(30.0)	42(30.0)	13.363	0.001
	Woman	201(56.1)	112(70.0)	98(70.0)		
Age (years)	60 ~ 69	143(39.9)	60(37.5)	57(40.7)	3.936	0.415
	70 ~ 79	182(50.8)	87(54.4)	64(45.7)		
	≥80	33(9.3)	13(8.1)	19(13.6)		
Marital status	Married	312(87.2)	133(83.1)	89(63.6)	37.124	<0.001
	Widowed	46(12.8)	27(16.9)	51(36.4)		
Income	<2000	34(9.5)	17(10.6)	7(5.0)	14.746	0.005
	2000 ~ 4,000	195(54.5)	77(48.1)	97(69.3)		
	>4,000	129(36.0)	66(41.3)	36(25.7)		
Residential mode	Living alone	16(4.5)	12(7.5)	29(20.7)	33.929	<0.001
	Non living alone	342(95.5)	148(92.5)	111(79.3)		
Education	Primary and below	40(11.2)	23(14.4)	21(15.0)	4.747	0.577
	Middle school	115(32.1)	45(28.1)	48(34.3)		
	High school	84(23.5)	42(26.2)	35(25.0)		
	College and above	119(33.2)	50(31.3)	36(25.7)		
Chronic disease status	None	136(38.0)	44(27.4)	58(41.4)	18.435	0.001
	One	148(41.3)	58(36.3)	43(30.7)		
	Two or more	74(20.7)	58(36.3)	39(27.9)		
Smoke	Yes	77(21.5)	38(23.8)	28(20.0)	0.640	0.726
	No	281(78.5)	122(76.2)	112(80.0)		
Alcohol	Yes	115(32.1)	47(29.4)	34(24.3)	89.409	<0.001
	No	243(67.9)	113(70.6)	106(75.7)		
Interests	Yes	220(61.5)	85(53.1)	69(49.3)	7.262	0.026
	None	138(38.5)	75(46.9)	71(50.7)		
Exercise	Almost never	53(14.8)	30(18.8)	35(25.0)	10.608	0.031
	Occasionally	264(73.7)	112(70.0)	83(59.3)		
	Often	41(11.5)	18(11.2)	22(15.7)		

### 3.6 Comparison of differences in cognitive function among elderly people in communities with different latent profiles of loneliness

The scores and total scores of various dimensions of cognitive function for elderly people in communities with different latent profiles of loneliness are shown in Table 3. Multiple comparisons of cognitive function scores were conducted, and the analysis results showed that there was a statistically significant difference (*p* < 0.05) in the total score of the Montreal Cognitive Assessment Scale among the three categories; There were statistically significant differences (*p* < 0.05) in the scores of naming, language, abstraction, and delayed recall dimensions among different categories.

**Table 3:** Comparison of differences in cognitive function among elderly people in communities with different latent profiles of loneliness (Score, $\bar{x}$ ± s).

Class	Visuospatial	Naming	Attention	Language	Abstraction	Memory	Orientation	Total
C1 (*n* = 358)	3.29 ± 1.12	2.96 ± 0.23	5.78 ± 0.50	2.79 ± 0.44	1.08 ± 0.65	3.60 ± 1.25	5.92 ± 0.35	25.42 ± 2.33
C2 (*n* = 160)	3.11 ± 1.21	2.96 ± 0.19	5.74 ± 0.59	2.72 ± 0.46	0.95 ± 0.57	3.26 ± 1.23	5.87 ± 0.43	24.61 ± 2.64
C3 (*n* = 140)	3.09 ± 1.22	2.88 ± 0.37	5.69 ± 0.65	2.68 ± 0.48	0.89 ± 0.62	3.21 ± 1.27	5.88 ± 0.41	24.31 ± 2.66
*F*	2.159	5.498	1.266	3.345	5.844	7.123	1.188	12.334
*P*	0.116	0.004	0.283	0.036	0.003	0.001	0.306	<0.001

### 3.7 Multivariate logistic regression analysis of latent profiles of loneliness among elderly people in the community

Logistic regression analysis was conducted with the potential category of loneliness among elderly people in the community as the dependent variable and the indicators with statistically significant differences in the univariate analysis as the independent variables. Logistic regression analysis showed that gender, chronic disease status, marital status, living style, presence of cognitive impairment, and depression status were potential influencing factors for loneliness among elderly people in the community (*p* < 0.05), as shown in Table 4.

**Table 4:** Multivariate logistic regression analysis of latent profiles of loneliness among elderly people in the community (*N* = 658).

Variable	*β*	*SE*	Wald *χ*^2^	*P*	OR	95%CI
C2 vs. C1*						
Intercept	−1.86	0.297	39.336			
Gender (woman)	0.859	0.283	9.196	0.002	2.360	1.355 ~ 4.110
Chronic disease status (two or more)	0.633	0.267	5.628	0.018	1.884	1.116 ~ 3.180
Depression	0.228	0.055	17.416	0.001	1.256	1.129 ~ 1.399
C3 vs. C1*						
Intercept	−2.69	0.347	60.158			
Marital (widowed)	0.818	0.343	5.697	0.017	2.266	1.158 ~ 4.437
Residential mode (living alone)	0.912	0.453	4.048	0.044	2.49	1.024 ~ 6.055
Cognitive impairment	0.527	0.253	4.348	0.037	1.693	1.032 ~ 2.778
Depression	0.336	0.057	35.165	0.001	1.399	1.252 ~ 1.563

C1* is a reference category.

## 4 Discussion

### 4.1 Heterogeneity of loneliness among elderly people in the community

From the standpoint of the “Life Course-Social Ecological Perspective,” the issue of loneliness among the elderly is investigated. This phenomenon is considered to be multi-faceted, as it is not only a psychological and emotional problem at the individual level, but also an unavoidable social problem (Cacioppo and Cacioppo, 2018).

As the findings indicate, the overall score of loneliness among elderly people in the community in this study is at a medium to low level, but there are still many elderly people in the community who have limited emotional communication with others and lack social activities. It is necessary to pay attention to the psychological status of elderly people in the community. This study adopts a latent profile analysis method to classify the loneliness of elderly people in the community. The results show that there are obvious classification characteristics of loneliness symptoms, which can be divided into three latent profiles: low loneliness type, social loneliness type, and emotional loneliness type. The fitting of various model indicators is good and in line with practical significance, indicating differences in loneliness among potential profiles, reflecting the heterogeneity of loneliness among elderly people in the community. This finding aligns with the core tenet of life course theory, which posits that variations in social relationships and accumulated resources experienced by individuals across different life stages contribute to a structural categorization of loneliness in later life (Elder, 1998). Farmer et al. (2022) found in their exploration of the current situation of social loneliness and medicine use among the elderly that social loneliness can be divided into five potential categories. Among them, the socially active group had the lowest drug use rate, while the group with less social activity and emotional loneliness had the highest medicine use rate. A longitudinal study on the analysis of loneliness transition found that groups with emotional loneliness are more likely to transition to the high loneliness group (Hammond et al., 2022), which may be due to the loneliness caused by insufficient emotional communication with others in the elderly, making it more difficult to fill in from social activities.

In this study, it was found that community elderly people in the emotional loneliness group scored higher in item 1 “Lack of companionship from others” and item 8 “Having someone by my side, but no one cares about me.” This group of people may have less emotional communication with family or friends, and the resulting loneliness not only increases the incidence of mental illnesses such as anxiety and depression (Wang et al., 2020). Therefore, appropriate emotional support and psychological counseling should be provided for this group of people. The social loneliness group scored higher in item 5 “I feel distant from others,” which may be related to the sense of gap after age and social role changes. Previous studies have found that for this group, more active community activities should be carried out to meet the respect and self realization of the elderly, and enrich their later life in the community (Hoang et al., 2022; Paquet et al., 2023). Using latent profile analysis to identify potential subtypes of loneliness among elderly people in the community, understanding the characteristics of loneliness among different groups, can help to carry out targeted health management and psychological counseling.

### 4.2 Factors influencing the latent profile of loneliness among elderly people in the community

Social-ecological systems theory posits that each individual is perpetually situated within a series of nested and interacting systems, and that the superimposition of micro-systems (e.g., at the family level), meso-systems (e.g., participation in community activities), and exosystems (e.g., culture) engenders varied forms of loneliness in older adults, owing to the differential levels of environmental support available to them as they confront the loss of their relationships (Vaezghasemi et al., 2023).

This study found that there were statistically significant differences (*p* < 0.05) in six variables, including gender, marital status, educational level, and interests, among elderly people in communities with different types of loneliness. These correspond to the micro, meso, and exosystems mentioned in the social-ecological system, which are key influences on the generation of heterogeneity. Compared with the low loneliness group, women and elderly people living in communities with multiple chronic diseases are more likely to belong to the social loneliness group. This may be due to the fact that men’s social networks may be more complex and their ability to handle negative emotions may be stronger (Moshtagh et al., 2022). In contrast, women who experience events such as the loss of a spouse or retirement during the aging process may undergo a more pronounced sense of disengagement from their familial roles, leading them to rely on kinship ties, such as spouses or children, to establish their social networks (Lu et al., 2023). The elderly with less social activity or lower frequency of social contact with others may experience negative emotions such as loneliness, but lower social support can affect their cognitive function. Meanwhile, low social support among the elderly can indirectly have adverse effects on individual cognitive function by affecting depressive emotions (Pais et al., 2021). Elderly people with multiple chronic diseases, such as joint pain, respiratory difficulties, etc., may limit physical mobility, which directly hinders offline social participation; and most of the elderly people will be difficult to take care of themselves due to the negative emotions of anxiety, reduce the frequency of interaction with the outside world, and at the same time, may be due to the stigmatization of the disease triggered by social anxiety (de la Torre-Luque et al., 2019), and indirectly caused by a lack of physical activity, which can cause physical and psychological double blow to the elderly people (Kandola et al., 2023).

Compared with the low loneliness group, community elderly people with marital status of no spouse, living alone, cognitive impairment, and high depression scores are more likely to belong to the emotional loneliness group. Research has found that different family structures have an impact on the level of loneliness (Losada-Baltar et al., 2021). Elderly people in widowed communities are more likely to live alone and receive less support and emotional feedback from within their families. The social support level of elderly people living alone is significantly lower than that of non-living alone elderly people, and their sense of loneliness is relatively high, which can easily associated with negative emotions such as depression (Widhowati et al., 2020). Therefore, community healthcare personnel identify different subtypes of loneliness among elderly people in the community through questionnaires, visits, and other forms during health checkups, in order to provide personalized health guidance.

For socially isolated elderly people, their level of social participation is relatively low. Understanding the interests and hobbies of the elderly can improve their relatively single social network, enhance their sense of belonging and social adaptability through group activities, and thus improve their quality of life and happiness (Fakoya et al., 2020). Group activities can increase positive emotions among elderly people living in nursing homes and reduce their experience of loneliness. On the other hand, for elderly people in emotionally lonely communities, not only should social support be provided, but also more attention should be paid to psychological counseling to meet their spiritual needs for love and belonging. To solve the problem of loneliness among the elderly, intervention measures should be adjusted to meet the needs of individuals and specific groups, and targeted interventions should be taken.

### 4.3 The relationship between the latent profile of loneliness among elderly people in the community and cognitive function

This study found that the detection rate of cognitive impairment among elderly people in the community was 31.00%, which is relatively high compared to previous research results (Bai et al., 2022; Rajan et al., 2021). This may be due to regional differences, residential patterns, and variations in the selection of evaluation tools. A cohort study found that there were statistically significant differences in the Montreal total score and dimensions scores for different levels of loneliness (Wang et al., 2022). The higher the level of loneliness, the higher the risk of mild cognitive impairment, and depression and anxiety may play a mediating role between the two. This study found that compared to elderly people in communities with low levels of loneliness, the detection rate of cognitive impairment in elderly people with social and emotional loneliness significantly increased. This result reflects a close correlation between high levels of loneliness and cognitive decline.

This study found significant differences in cognitive function and sub dimension scores among different categories, but the risk of cognitive impairment in elderly individuals with emotional loneliness was significantly higher than the other two categories (*OR* = 1.693). Compared to individuals with low levels of loneliness and social isolation, older adults with emotional loneliness who show less or do not receive timely feedback have a more severe impact on cognitive function. The analysis results show that emotionally lonely elderly people who are widowed or living alone have a higher proportion and often have less social participation, which may associated with negative behavior patterns and negative emotions such as depression and loneliness (Oughli and Lee, 2024). From a life course perspective, the high proportion of widowhood and loneliness in this group reflects the cumulative loss of intimacy. In a study by Oken et al. (2024) it was found that persistent loneliness activated the HPA axis triggering chronic inflammation and direct damage to the hippocampus, further demonstrating the close association between high loneliness levels and cognitive function. In this study, the higher the depression score, the more likely it was to belong to the emotional loneliness group (OR = 1.399), and depression often interacts with feelings of loneliness and cognitive impairment (Ferri et al., 2021; McHugh et al., 2020). Previous studies have found that psychological problems such as depression may associated with cognitive impairment through immune regulatory pathways (Chen et al., 2023). Therefore, community healthcare workers should pay more attention to emotionally lonely elderly people when preventing cognitive impairment in the elderly, understand their psychological status, and provide targeted health management to improve their cognitive function.

## 5 Conclusion and limitation

This study divides the loneliness of elderly people in the community into three types through potential profile analysis: low loneliness type, social loneliness type, and emotional loneliness type. Among them, the prevalence of cognitive impairment was higher in the emotional loneliness group. The contribution of this study is to confirm that the type of emotional loneliness is more specific than the degree of loneliness in predicting the risk of cognitive impairment in Chinese community-dwelling older adults, which suggests that community healthcare workers should pay more attention to this group of older adults, and that the community or the clinic can adopt a type-specific and precise intervention rather than a simple socialization promotion. This finding suggests that community or clinical interventions may be more appropriate for this group of older adults, and that retroactive problem solving may be more effective in improving cognitive functioning. By identifying the characteristics of elderly people, understanding their types of loneliness, and taking effective intervention measures to help alleviate their own loneliness, cognitive function can be improved, and their happiness and quality of life can be enhanced.

In addition, the cross-sectional survey method adopted in this study can be used to explore the trajectory changes of various categories of loneliness and their relationship with cognitive function through longitudinal research in the future. The main questionnaire used in this study is subjective, with less objective data and regional sampling restrictions. In the future, the sample size can be expanded and objective measurement indicators can be increased.

## Data Availability

The original contributions presented in the study are included in the article/supplementary material, further inquiries can be directed to the corresponding author.
